# Peripheral Nervous System Pain Modulation

**DOI:** 10.2174/1570159X21666230803100400

**Published:** 2023-08-04

**Authors:** Marcin Karcz, Christopher Gharibo

**Affiliations:** 1 Division of Pain Medicine, Department of Anesthesia, New York University Grossman School of Medicine, New York, NY, USA

**Keywords:** Peripheral nerve stimulation, acute pain, chronic pain, modulation, neuromodulation, mechanisms

## Abstract

The percutaneous technique of electrode insertion in the vicinity of the greater occipital nerves to treat occipital neuralgia was first described in the 1990s by Weiner and Reed. This subsequently stimulated awareness of peripheral nerve stimulation (PNS). The more recent advent emergence of a minimally invasive percutaneous approach by way of using ultrasound has further increased the interest in PNS as a viable alternative to more invasive techniques. PNS has become more popular recently and is increasingly used to treat various pain conditions. Its foundation is fundamentally based on the gate control theory, although the precise mechanism underlying its analgesic effect is still indefinite. Studies have demonstrated the peripheral and central analgesic mechanisms of PNS by modulating the inflammatory pathways, the autonomic nervous system, the endogenous pain inhibition pathways, and the involvement of the cortical and subcortical areas. Peripheral nerve stimulation exhibits its neuromodulatory effect both peripherally and centrally. Further understanding of the modulation of PNS mechanisms can help guide stimulation approaches and parameters to optimize the use of PNS. his chapter aims to review the background and mechanisms of PNS modulation. PNS is becoming one of the most diverse therapies in neuromodulation due to rapid evolution and expansion. It is an attractive option for clinicians due to the simplicity and versatility of procedures that can be combined with other neuromodulation treatments or used alone. It has a distinct role in the modulation of functional conditions.

## 1 INTRODUCTION

The regulation of pain signaling pathways along the central and peripheral nervous system that mediate excitatory or inhibitory responses is referred to as pain modulation [1].

Peripheral nerve stimulation is based on the method of orthodromic stimulation of non-nociceptive Aβ nerve fibers. There is an excitation of respective dorsal horn interneurons that are involved in the processing and transmitting nociceptive information *via* peripheral Aβ and C nerve fibers by activation of these fibers. Nonpainful stimulation of the peripheral nerve territory results in decreased pain signals. Although the modulation of pain can be elicited by targeting several nociceptors along the transmission pathway, this chapter focuses on mechanisms of peripheral nervous system modulation.

## 2 PERIPHERAL NERVE NEUROMODULATION

It is believed that PNS represents the first clinical application of the gate control theory initially proposed by Melzack and Wall. This was when Wall and Sweet applied stimulation to their trigeminal nerve branches to study the effects of stimulation on evoked pain [2]. Following this, spinal cord stimulation (SCS) dominated the field of neurostimulation. PNS therapy was used in several centers with the expertise and performed initially by surgical dissection. This involved a direct identification of the target nerve followed by fascial graft harvest and subsequently by electrode placement [3]. The targeted initial nerves were either in the upper or lower extremity. The most common pathology for stimulation was CRPS-II, surgical scarring, and trauma cases [3]. This technique of PNS has been succeeded by less invasive methods of placing electrodes.

TENS and acupuncture are two common practices that have been used traditionally to treat pain [4]. The technique of PNS is often compared with these practices. Acupuncture is based on the use of needles to stimulate the nerves at specific points in the body. Using needles, cutaneous signals of low frequency are conducted to the spinal cord. This manifests as an interaction between these visceral and peripheral inputs. There is subsequently both inhibition of central sensitization and peripheral neuromodulation [4]. TENS uses transcutaneous electrodes to apply varying stimulus intensities to the peripheral nerve, thereby causing neuromodulation at both central and peripheral levels. PNS similarly involves subcutaneous nerve stimulation in the painful region using the same concept as TENS [5].

The aim of percutaneous electrical nerve stimulation (PENS) is to relieve pain by targeting specific peripheral nerves using electrical stimulation. It involves the insertion of fine needles near the affected nerves into the skin and the stimulation of the nerves by the passage of an electrical current through the needles [6]. PENS aims to relieve unrelenting, chronic pain by the stimulation of peripheral sensory nerves. The technique of PENS involves the insertion of needle-based electrodes rather than lead implantation with the emission of low-voltage currents to desensitize the nerve endings in the region of pain.

PENS, similarly to TENS, targets the cutaneous, terminal fibers of sensory nerves in the skin. And it, however, reduces the resistance to stimulation seen with TENS by using microneedles to penetrate the exterior layers of the skin [7, 8]. Unlike PNS, PENS lacks the diverse, prospective data that support its use. The benefit of PENS is that it may offer pain relief to patients in situations where surgery may be contraindicated and overall does not require detailed anatomical knowledge and is much less complex than PNS [9].

PNS and spinal cord stimulation (SCS) are both neuromodulation systems that deliver electrical pulses to the peripheral nerves and spinal cord, thus changing the transmission of pain signals to the brain. The effects of this stimulation on nociceptive processing, as well as spinal inhibition, are shown in Fig. (**1**) [10].

The large diameter non-nociceptive Aβ-fibers in the dorsal column are directly stimulated by conventional SCS. Subsequently, there is antidromic inhibition of those nociceptive signals entering the dorsal horn [11]. There is also orthodromic propagation of electrical impulses along the non-nociceptive nerve fibers [11]. The activation of supraspinal pain processing systems, such as the thalamic centrum medianum and the pretectal nucleus, are ways that SCS can modulate pain perception. Evidence has shown that activity related to pain transmission in supraspinal areas can be inhibited by electrical stimulation. These areas include the reticular formation (RF), the periaqueductal gray (PAG), the locus coeruleus (LC), and the rostral ventromedial medulla (RVM), [12].

## 3 PERIPHERAL NERVE STIMULATION: MECHANISM OF ACTION

Amplified transmission of the pain signals to the sensory cortex, and thalamus is the result of changes in the descending variation from the rostral ventromedial medulla and the periaqueductal gray. Aberrant sensory processing is subsequently the result of expanding the pain representation in the cortex [13]. The interaction of several different components, including the excitation of interneurons, ectopic firing, and glial activation, act at both supraspinal and spinal levels leading to an abnormal nociceptive signal processing, maintenance of a hyperexcitable state and decreased threshold at downstream peripheral levels [14].

The exact mechanism of PNS accountable for pain relief is unknown. Still, it is assumed to be likely due to a conduction block in small-diameter afferent fibers, thus preventing the arrival of nociceptive information to the central nervous system. Additionally, there may be a stimulation-induced blockade of cell membrane depolarization, causing suppression of dorsal horn activity and a reduction in the excitation of C-fiber nociceptors and hyperexcitability [15]. Furthermore, there may be potentiation of dorsal horn neurons as well as the release of inhibitory neurotransmitters such as GABA and simultaneously a depletion of excitatory amino acids, such as glutamate and aspartate [16].

## 4 PERIPHERAL NERVE STIMULATION: THE PERIPHERAL PATHWAY MECHANISM

There is increased blood flow that arises from the release of local mediators such as prostaglandins and endorphins during chronic pain arising from peripheral nerves [17]. These inflammatory mediators and blood flow are downregulated at the peripheral level by the PNS [17].

High-frequency stimulation has been shown to can cause an exponential decline in the conduction velocity of both non-myelinated and myelinated nerve fibers [18]. Furthermore, the conduction velocity and excitability of nerve fibers following tetanic stimulus appear to be subnormal [18]. This can be seen clinically as an excitation failure of A and C fibers due to repeated electrical stimulation of intact radial and saphenous nerves [18].

The mechanism of action for peripheral nerve stimulation may be more likely demonstrated to be of a central origin since stimulus intensities above the threshold of perception but below the threshold for pain are responsible for the analgesic effect of PNS [19].

## 5 PERIPHERAL NERVE STIMULATION: THE CENTRAL PATHWAY MECHANISM

As described previously, the PNS may be involved in modulating higher CNS foci, including the somatosensory cortex, anterior cingulate cortex, parahippocampal areas, and the dorsal lateral prefrontal cortex [20]. Additionally, the PNS inhibits A-delta and C fibers and activates A beta fibers in the periphery, leading to inhibition of dorsal interneurons consistent with the gate control theory [19, 20].

Thus, at the spinal level, there are GABA and glycine-mediated increases in serotonin and dopamine metabolites and changes in the levels of the calcitonin gene-related protein (CGRP) and substance P. There are also anatomic changes in the medial lemniscus pathway and spinothalamic tracts, including a widespread inhibition of dorsal wide dynamic range neurons, with decreased A beta fiber activation [21].

Furthermore, the trigeminocervical complex and the nociceptive inflow to second-order neurons in the spinal cord are subject to modulation by descending inhibitory projects from the brain stem structures. These structures include the nucleus raphe magnus, rostroventral medulla, and periaqueductal gray (PAG). Stimulation of these regions produces widespread antinociception [21].

## 6 APPLICATIONS OF PERIPHERAL NERVE STIMULATION

Electroanalgesia gained traction with the advent of centrally-acting transcutaneous electrical nerve stimulation (TENS). The neuromodulatory effect of electroanalgesia was first described by Melzack and Wall with the gate control theory; this phenomenon describes the dampening of nociceptive signals from A∂ and C fibers by interference from non-nociceptive sensory signals transmitted by large-diameter Aß fibers *via* dorsal horn interneurons [22].

Furthering this theory, Torebjork and Hallin recruited six healthy males to participate in a study involving repeated electrical stimulation of the A∂ and C fibers in intact radial and saphenous nerves resulting in excitation failure [23]. Another theorized mechanism of peripheral nerve stimulation (PNS)-induced pain modulation is a biochemical alteration of microenvironments *via* the downregulation of excitatory neurotransmitters and inflammatory mediators and decreased ectopic discharges and Wallerian degeneration [23].

In clinical practice, PNS was typically reserved for patients with chronic, intractable focal pain who failed conventional treatment modalities with at least some sensation intact (to recruit Aß fibers) [24]. This may be attributed to conventional, more invasive, and permanently implanted PNS systems that require operator expertise with technically challenging instruments and dedicated hardware. In recent times, ultrasound-guided percutaneous lead implantation along targeted nerves and increasing practitioner experience have made PNS a more favorable approach [25].

## 7 PNS IN THE MANAGEMENT OF ACUTE PAIN

Ilfeld and Finneran recently conducted a randomized, controlled, multicenter trial to determine the efficacy of percutaneous PNS on acute postoperative pain and its relationship to cumulative opioid consumption (measured in oral morphine equivalents). Participants underwent preoperative electrical lead implantation and single-shot long-acting local anesthetic injection with the following targets: sciatic nerve for major foot/ankle surgery, femoral nerve for anterior cruciate ligament reconstruction, and brachial plexus for rotator cuff repair. Postoperatively, participants were randomized to 2 weeks of electrical or sham stimulation. The results showeddecreased postoperative opioid consumption (*p* < 0.001) in the PNS group (average 5 mg; interquartile range 0 to 30 mg) compared to the sham group (average 48 mg; interquartile range 25 to 90 mg); similarly, there was a significant difference between pain intensity scores (score between 0-10; *p* < 0.001) between the PNS group (1.1 ± 1.1) and sham group (3.1 ± 1.7) [26]. Furthermore, unlike peripheral nerve blocks that induce motor, sensory, and often proprioceptive deficits, leading to increased risk of falls and decreased participation in physical therapy, PNS was not associated with similar side effects [27].

Another study investigated the efficacy of ultrasound-guided percutaneous PNS in hallux valgus osteotomies. Current analgesic modalities for these surgeries include a combination of oral medications, surgical infiltration with local anesthesia, and single-shot *versus* continuous peripheral nerve block. However, these interventions are limited by pain reemergence; with continuous peripheral nerve blocks, limitations also include catheter infection/dislodgement/ leaking, especially if left implanted for more than 3 to 4 days, the bulkiness of the apparatus, and cost and availability of infusion pumps in the outpatient setting. In this pilot study, electrical leads were preoperatively implanted along the sciatic nerve between the subgluteal region and bifurcation in patients undergoing foot surgery [28]. Participants were randomized to receive 5 minutes of PNS or sham followed by a 5-minute crossover followed by 30 minutes of continuous stimulation. Leads were extracted with simple traction between 2 to 4 weeks postoperatively. Results showed a downward pain trajectory in patients randomized to initial PNS; in contrast, patients randomized to initial sham treatment did not show this trajectory until after crossover to PNS. In both groups, pain scores decreased by > 50% in the subsequent continuous stimulation period [29]. The researchers demonstrated similar outcomes with femoral PNS in participants undergoing total knee arthroplasty [30].

There is a paucity of literature investigating the use of peripheral neurostimulation in acute pain. Nevertheless, these studies suggest the integration of PNS into the acute pain treatment model to reduce opiate consumption, minimize opioid-related adverse effects and even increase direct home discharges. The benefits of PNS in the operative setting appear most impactful preoperatively to decrease baseline pain, which is associated with greater postoperative pain [31].

## 8 PNS IN THE MANAGEMENT OF CHRONIC PAIN

The first documented application of PNS was of the greater occipital nerve (leading to inhibition of nociceptive processing at the spinal trigeminal nucleus) for the management of intractable headaches in 1999 [32]. Dodick *et al.* [33] expanded upon the safety and efficacy profile of this procedure in their randomized, multicenter, double-blinded 52-weeks-long study. In the study, 150 patients with intractable chronic migraine were randomized to sham/control *versus* stimulation/active treatment for 12 weeks, followed by 40 weeks of active treatment for all participants. Outcomes measured included pain intensity, number of headache days, migraine disability assessment (MIDAS), Zung Pain and Distress (PAD), quality of life, satisfaction, adverse events, and direct reports of headache pain relief. There was a significant reduction in number of headache days (6.7 days ± 8.4 days; *p* < 0.001) reflected in the results. Pain intensity was reduced by 50% in nearly 50% of participants. Additionally, 65% reported good or excellent headache relief, and more than 50% reported satisfaction. Although these findings are promising, 70% of participants experienced device-related adverse events, including battery failure, lead migration/breakage, allergic reaction, infection and wound site complications, and undesirable changes in stimulation; of those, 8.6% required hospitalization, and 40.7% required surgical intervention. In summation, this study pioneered the concept of long-term analgesia with PNS while simultaneously paving the way for further investigation into safety.

A prospective, multicenter trial investigated the utility of PNS as a safe, minimally invasive, nondestructive, motor-sparing alternative to repeat radiofrequency ablation in patients with chronic back axial pain. The rationale for this study was to minimize undesirable outcomes associated with lumbar radiofrequency ablation, including recurrence of pain, obliteration of medial branch nerves and associated denervation atrophy of the multifidus, and diminishing returns after repeat ablation. The investigators recruited patients with chronic axial low back pain who failed traditional analgesics therapies and experienced recurrence of pain after ablation; these patients subsequently underwent PNS lead implantation targeting the medial branch nerves and PNS for up to 60 days (at which the leads were extracted). The researchers reported a significant reduction in pain intensity (meaningful outcome measured as ≥ 50% reduction in BPI-5 scores; 6.3 at baseline to 2.4; *p* < 0.0001), disability (meaningful outcome measured as > 10-point reduction in ODI; mean 21-point reduction; *p* < 0.002), and pain interference (meaningful outcome measured as ≥ 30% reduction in BPI-9 scores; mean 61% reduction; *p* < 0.0001) 2 months after the intervention. The results continued in this trajectory at the 5-month interval as well (pain intensity BPI-5 score 3.1, *p* < 0.0001; disability ODI score 26.1, *p* = 0.003; pain interference BPI-9 score 3.2, *p* = 0.0016) [34]. The outcomes of this study have subsequently been extrapolated to treat other chronic pain conditions, including complex fibromyalgia [35], migraine and chronic daily headaches, and inguinal and genital pain syndromes.

PNS may also exert central effects. Recent studies have suggested PNS reduces Aß fiber activity in the medial lemniscal tract and inhibits wide dynamic range neurons in the dorsal horn, thus leading to decreased central sensitizations and hyperalgesia [36]; furthermore, animal models have shown that PNS causes regulatory changes along serotonergic, GABAergic, and glycinergic pathways along the spinal cord [37]. Newer studies have shown PNS may have a role in the neuromodulation of centrally-mediated phantom limb pain and complex regional pain syndrome (CRPS) [38]. In a double-blind, placebo-controlled randomized study, 28 lower extremity amputees with postamputation pain were enrolled to be randomized to placebo or percutaneous femoral and sciatic PNS for 4 weeks; the placebo group then crossed, and all participants received an additional 4 weeks of PNS. The results showed 58% of participants in the PNS reported ≥ 50% reduction in average postamputation pain during weeks 1-4, as compared to only 14% in the placebo group (*p* = 0.037). This trend continued at 8 weeks, with 67% of participants in the PNS reported ≥ 50% reduction in average postamputation pain, as compared to only 14% in the placebo group (*p* = 0.014) [39]. The authors concluded activation of large-diameter sensory afferents *via* PNS attenuates peripheral nociceptive signals and amplifies tactile and proprioceptive afferents to recondition the maladaptive cortical reorganization that occurs with amputation. Similarly, a case series reported on 12 patients with upper limb CRPS who underwent brachial plexus neuromodulation. After a trial phase, 10 patients reported ≥ 50% pain reduction and underwent permanent implantation. At the 1-year follow-up, all 10 patients reported significant improvement in pain across a variety of pain measures, including the visual acuity scale (57.4% ± 10%; *p* = 0.005), neuropathic pain scale (60.2% ± 12.9%; *p* = 0.006), and SF-12 physical and mental scale (21.9% ± 5.9%; *p* = 0.015) [40]. These studies suggest a promising role for PNS in the reversal of centrally-mediated pain conditions.

Somewhat like PNS, electroacupuncture applies low current and low-frequency signals to modulate pain processing by the peripheral nervous system. The mechanism is thought to be secondary to the activation of the sympathetic nervous system, which in turn increases leukocyte adhesion, stimulates opioid release from fibroblasts and adrenergic receptors, inhibits COX2 and other local inflammatory mediators, and upregulates cannabinoid receptors [41].

## CONCLUSION

Chronic pain is responsible for significant morbidity worldwide, causing extreme interference with daily activities, limitations to mobility, psychiatric illness, and dependence on opioid analgesics in selective patient populations. PNS has been regaining popularity as an effective treatment modality due to a need for establishing an efficient treatment mechanism as well as the growing concerns about the potential hazards of currently prescribed pain medications. PNS is rapidly becoming an established treatment approach for both acute and chronic pain. Its early origin is based on the gate control theory and a central mechanism. PNS’ peripheral and central analgesic mechanisms by the modulation of cortical and subcortical area' inflammatory pathways have been described in several studies. Furthermore, it has been shown to act at the level of the endogenous pain inhibition pathways as well as the autonomic nervous system. Pain modulation can also be elicited by targeting several nociceptors along the transmission pathway. This chapter has focused on these mechanisms of peripheral nervous system modulation. We believe that an understanding of the modulatory mechanisms of PNS can help guide the approach to optimizing the use of PNS.

## Figures and Tables

**Fig. (1):**
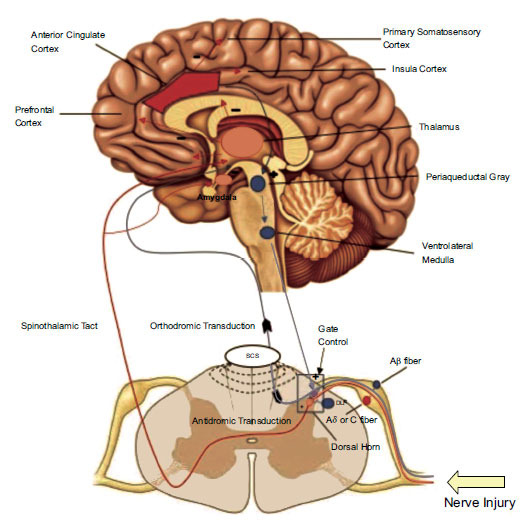
Graphic representation showing the consequences of peripheral nerve and spinal cord stimulation on nociceptive processing, including cortical modulation, activation of descending inhibitory system, and segmental spinal inhibition [12].

## LIST OF ABBREVIATIONS

CGRP: Calcitonin Gene-related Protein
CRPS: Complex Regional Pain Syndrome
LC: Locus Coeruleus
PAG: Periaqueductal Gray
PENS: Percutaneous Electrical Nerve Stimulation
RF: Reticular Formation
RVM: Rostral Ventromedial Medulla
SCS: Spinal Cord Stimulation
TENS: Transcutaneous Electrical Nerve Stimulation
